# Effects of acute ingestion of a multi-ingredient pre-workout supplement on lower body power and anaerobic sprint performance

**DOI:** 10.1186/1550-2783-12-S1-P49

**Published:** 2015-09-21

**Authors:** AR Jagim, G Wright, K Schultz, C St Antoine, MT Jones, J M Oliver

**Affiliations:** 1 Exercise & Sport Science Department, University of Wisconsin - La Crosse, La Crosse, WI, 54603, USA; 2 Division of Health and Human Performance, George Mason University, Fairfax, VA, 22030, USA; 3 Kinesiology Department, Texas Christian University, Fort Worth, TX, 76129, USA

## Background

Multi-ingredient pre-workout supplements (MIPS) are becoming popular dietary supplements among strength and power athletes. These products frequently include caffeine, creatine, beta-alanine, and branched-chain amino acids as the primary ingredients. When studied on an individual basis, several of these ingredients have been shown to increase muscular power following acute ingestion; however, little is known in regard to a synergistic effect when said ingredients are combined. The purpose of this study was to determine if short-term, MIPS ingestion influences muscular power and anaerobic sprint performance.

## Methods

In a double-blind, randomized, and crossover design; 12 Division III male, football players (18.8 ± 1.2 yrs; 180 ± 12 cm; 89.3 ± 11 kg; 13.6 ± 4.9% BF) completed one baseline session and two subsequent testing sessions to determine the efficacy of acute ingestion of a MIPS. The initial baseline session consisted of body composition assessment and familiarization with the jump mat and non-motorized force treadmill. In testing Session 1, participants ingested either 1 serving of a commercially available MIPS (SUP) that contained 4g of carbohydrates, 2g of creatine hydrochloride, 3g of beta-alanine, 1.5g of betaine, 1g of taurine, 600mg of N-acetyl L-Cysteine, 150mg of Alpha-Glyceryl Phoshporyl Choline, 6g of citruline malate, 500mg of beet extract, 6g of BCAA's, 1.5g of L-tyrosine, 300mg of caffeine anhydrous, 50mcg of huperzine A and 5mg of BioPerine; or a placebo (PLA). Following a post-consumption 30-minute waiting period, participants completed a warm-up of 10 body weight exercises. Next, they completed a counter-movement vertical jump (CMVJ) test on a jump mat (*Just Jump System, Probotics, AL, USA*), which consisted of three attempts with the highest CMVJ being recorded for analysis and converted to power (W) using previously described methods [1]. Following the CMVJ, participants completed a 25-second maximal effort sprint test on a non-motorized force treadmill with the resistance set at 18% of their bodyweight. Session 2 followed a week later in which participants repeated the testing protocol under the opposite treatment condition (SUP or PLA).

## Results

Mean values for CMVJ power and treadmill performance work under each treatment are included in Table 1. There were no significant differences in lower body peak (p = 0.584) or mean power (p = 0.584) as determined by CMVJ. A significant increase in mean power was observed in the MIPS condition (p = 0.034) during the anaerobic sprint test. No significant differences were observed for any of the remaining anaerobic sprint performance variables.

**Table 1:** 

Variable	SUP	PLA	p value
Peak Power (W)	1934 ± 379	1918 ± 376	0.719

Mean Power (W)	1468 ± 304	1397 ± 257	0.034*

Total Work (m)	107.1 ± 4.8	106.7 ±5.3	0.384

CMVJ (cm)	65.2 ± 7.0	65.8 ± 8	0.584

Peak Power (W)	6470 ± 895	6513 ± 898	0.584

Mean Power (W)	3415 ± 487	3438 ± 483	0.584

Values are presented as Mean±SD

*Significant difference between treatment conditions (p < 0.05).

## Conclusions

Results suggest that acute ingestion of a MIPS 30 minutes pre-exercise has no impact on lower body muscular power, but improves mean power output during a maximal-effort anaerobic sprint. Based upon the results of the current study, ingesting a MIPS prior to a training session may improve anaerobic capacity during bouts of exercise lasting < 30 seconds.
